# On the Action of the Pancreatic Fluid

**Published:** 1849-01

**Authors:** Ch. Bernard


*On the Action of the Pancreatic Fluid.* By M. Ch. Bernard.—The author of this paper concludes from his experiments that the pancreatic secretion is essential to the reception of fatty matters into the system. He found that it immediately produces an emulsion, when mixed with oily substances; a property which is not possessed by any other animal substance. The first action seems purely mechanical; but after a time a further change takes place, the fats being decomposed into their fatty acids and glycerine. In this state the bile, which does not act on the neutral fats, will readily take them up; and thus a mixture of bile and pancreatic juice, such as is found in the duodenum, has the double power of dissolving the neutral fats and the fatty acids. The author has found that if the pancreatic ducts be tied, no fatty matters find their way into the chyle.—*Ibid, from L'Institut*, Mai 3, 1848.

